# Clarifying the nature of stochastic fluctuations and accumulation processes in spontaneous movements

**DOI:** 10.3389/fpsyg.2023.1271180

**Published:** 2023-10-12

**Authors:** Carsten Bogler, Bojana Grujičić, John-Dylan Haynes

**Affiliations:** ^1^Bernstein Center for Computational Neuroscience, Charité-Universitätsmedizin Berlin, Berlin, Germany; ^2^Max Planck School of Cognition, Leipzig, Germany; ^3^Berlin School of Mind and Brain, Humboldt-Universität zu Berlin, Berlin, Germany; ^4^Department of Science and Technology Studies, University College London, London, United Kingdom; ^5^Berlin Center for Advanced Neuroimaging, Charité-Universitätsmedizin Berlin, Berlin, Germany; ^6^Clinic of Neurology, Charité-Universitätsmedizin Berlin, Berlin, Germany; ^7^Institute of Psychology, Humboldt-Universität zu Berlin, Berlin, Germany; ^8^Cluster of Excellence “Science of Intelligence”, Berlin Institute of Technology, Berlin, Germany

**Keywords:** Libet, spontaneous movement, accumulator models, stochastic fluctuations, drift diffusion model (DDM), readiness potential (RP)

## Abstract

Experiments on choice-predictive brain signals have played an important role in the debate on free will. In a seminal study, Benjamin Libet and colleagues found that a negative-going EEG signal, the readiness potential (RP), can be observed over motor-related brain regions even hundreds of ms before the time of the conscious decision to move. If the early onset of the readiness potential is taken as an indicator of the “brain’s decision to move” this could mean that this decision is made early, by unconscious brain activity, rather than later, at the time when the subject believes to have decided. However, an alternative kind of interpretation, involving ongoing stochastic fluctuations, has recently been brought to light. This stochastic decision model (SDM) takes its inspiration from leaky accumulator models of perceptual decision making. It suggests that the RP originates from an accumulation of ongoing stochastic fluctuations. In this view, the decision happens only at a much later stage when an accumulated noisy signal (plus imperative) reaches a threshold. Here, we clarify a number of confusions regarding both the evidence for the stochastic decision model as well as the interpretation that it offers. We will explore several points that we feel are in need of clarification: (a) the empirical evidence for the role of stochastic fluctuations is so far only indirect; (b) the interpretation of animal studies is unclear; (c) a model that is deterministic during the accumulation stage can explain the data in a similar way; (d) the primary focus in the literature has been on the role of random fluctuations whereas the deterministic aspects of the model have been largely ignored; (e) contrary to the original interpretation, the deterministic component of the model is quantitatively the dominant input into the accumulator; and finally (f) there is confusion regarding the role of “imperative” in the SDM and its link to “evidence” in perceptual decision making. Our aim is not to rehabilitate the role of the RP in the free will debate. Rather we aim to address some confusions regarding the evidence for accumulators playing a role in these preparatory brain processes.

## Introduction

Throughout the day, we have to make a multitude of decisions about *external* stimuli. For example, when we see a car crossing our lane on the highway, we step on the break to avoid a collision. An important factor is the level or quality of sensory information. For example, when driving in broad daylight we instantly see the dangerous car. But when it is foggy, we might be uncertain about whether it is a car or just a random pattern in the mist. In that case, we might need to look at the pattern for a bit longer and gather evidence across time. A popular approach for explaining perceptual decision making (PDM) under such varying levels of sensory evidence is the accumulator model (Smith and Ratcliff, 2004). It formulates a mechanism that accumulates sensory evidence across time and thus gradually improves the accuracy of a sensory decision. When the buildup of evidence crosses a set threshold the decision is reached, and a reaction can be triggered. Most accumulator models involve two key variables that are combined in an additive fashion: the first term is the constant component of the sensory evidence in each time step that reflects the mean evidence in each sample of information about the external stimulus; the second term is a noise term that accounts for the variability originating from fluctuations in external sensory evidence as well as from internal sources (note that for, e.g., a random dot kinematogram the sensory evidence can fluctuate from moment to moment; Purcell and Palmeri, 2017). The noise term accounts for the differences in response times. The accumulator adds both terms, the evidence and the variable term, as inputs to its ongoing total evidence tally. So, both the constant as well as the fluctuations contribute to the decision. When the external information is high (as in broad daylight) the process is dominated by the evidence, and the threshold can be reached quickly. When the external information is low (as in fog) the process is dominated by noise, and it takes longer to reach a decision. The model may also include a leak term so that the total evidence slowly decays if it is not refreshed, in which case the model is called a leaky stochastic accumulator or an Ornstein-Uhlenbeck process (Usher and McClelland, 2001).

In recent years, this approach has also been used to explain the neural mechanisms underlying simple, spontaneous voluntary actions (Schurger et al., 2012; Schurger, 2018). These movement decisions have been met with considerable interest in debates about free-will and volition (Libet, 1985). This is because such spontaneous decisions are preceded by a slow negative-going EEG signal, the so-called readiness potential (RP; Kornhuber and Deecke, 1965) that appears to occur even before the time at which a person reports to have made a conscious decision to move (Libet et al., 1983). To give a very rough summary, a debate has centered on the following notion: if the brain “knows” that a decision will occur before a participant has consciously made up their mind, then this might mean that the decision has happened before the conscious mind became involved, which has been debated as a potential challenge to conscious free will (for discussions, see Libet, 1985; Schurger et al., 2012, 2021; Brass et al., 2019). Here, we will not be interested in the free will debate, but in the mechanisms that occur before a self-initiated movement. According to the original interpretation, the onset of the RP is a “post-decisional” signal, meaning that the buildup begins only after the decision to move has been made by the brain. In that view, the early onset of the RP reflects an early decision of the brain that happens before consciousness kicks in Schurger et al. (2021).

Recently, an alternative kind of explanation has been proposed that is based on ongoing slow random fluctuations in brain activity and that places the decision at a much later time. As we will see, a key difference here is that the RP, rather than being post-decisional, reflects a pre-decisional stage where the decision has not yet been made and during which random fluctuations play a role in determining the precise time at which the threshold is reached. The stochastic decision model (SDM) was proposed by Schurger et al. (2012) and takes its inspiration from the abovementioned leaky accumulator model from perceptual decision making (Usher and McClelland, 2001). However, now the noise term from the accumulator takes center stage. The idea is that the decision is determined largely by the accumulation of random internal fluctuations. Fluctuation-based accounts have long been used to explain Libet’s findings (Eccles, 1985; Libet, 1985; Ringo, 1985; Stamm, 1985). These older accounts (some of which are dualist) do not explicitly employ accumulators, but slowly fluctuating signals.^1^

In the original version of the SDM (Schurger et al., 2012), two different sources provide input to a leaky accumulator. One input stems from noise fluctuations. The parameters that best fit the empirical data (specifically the shape of the RP and the distribution of waiting times) were such that the noise fluctuations alone did not drive the signal over the threshold within the typical time taken by participants to make a decision (i.e., the model would not have accounted for the distribution of waiting times). So, a second input (the “imperative”) is used that brings the process into the operating range close to the decision bound so that the accumulated internal fluctuations can spuriously drive the signal across the boundary. Or in the original words of the authors:

“*In our model this solution amounts to simply shifting premotor activation up closer to the threshold for initiation of the instructed movement and waiting for a random threshold-crossing event*” (Schurger et al., 2012, p. E2905).

In this version of the SDM, the process that drives the signal closer to the threshold is a constant input called an “urgency” or “imperative” signal, as it reflects the demand or imperative to move (Schurger et al., 2012). Depending on where the threshold is set, it is necessary because it prevents having to wait for a very long time for the decision (see above and below). Interestingly, this imperative signal is mathematically equivalent to the “evidence” signal in perceptual decision making, but it has a very different interpretation (see also below). Note that the word “urgency” used in Schurger et al. (2012) may have been confusing because of the different way that same word is sometimes used in the perceptual decision-making literature (Cisek et al., 2009). We thus follow Schurger (2018) and have replaced it with the term “imperative” here. Please note that within the broader modeling framework there are multiple ways to account for the urgency of a decision, for example using collapsing bounds (Hawkins et al., 2015), as well as multiplicative signals (see, e.g., Ditterich, 2006).

In the following, our primary aim is to clarify several points regarding the SDM that have led to confusions in the literature. While many authors correctly cite and discuss the architecture and the implications of the model, there still seem to be a lot of misunderstandings regarding several aspects. Our interest here is not to fully review the literature on the readiness potential, to re-introduce the readiness potential into the debate on free will, or to rule out the accumulator model as a potential mechanism for spontaneous actions. We will focus on the discussion of the mechanisms involved in movement initiation rather than the question of subjective experiences of volition. Our primary aim is to delineate more clearly what the SDM-related findings mean and what they do not mean.

## How does the accumulator model work?

**Figure 1:**
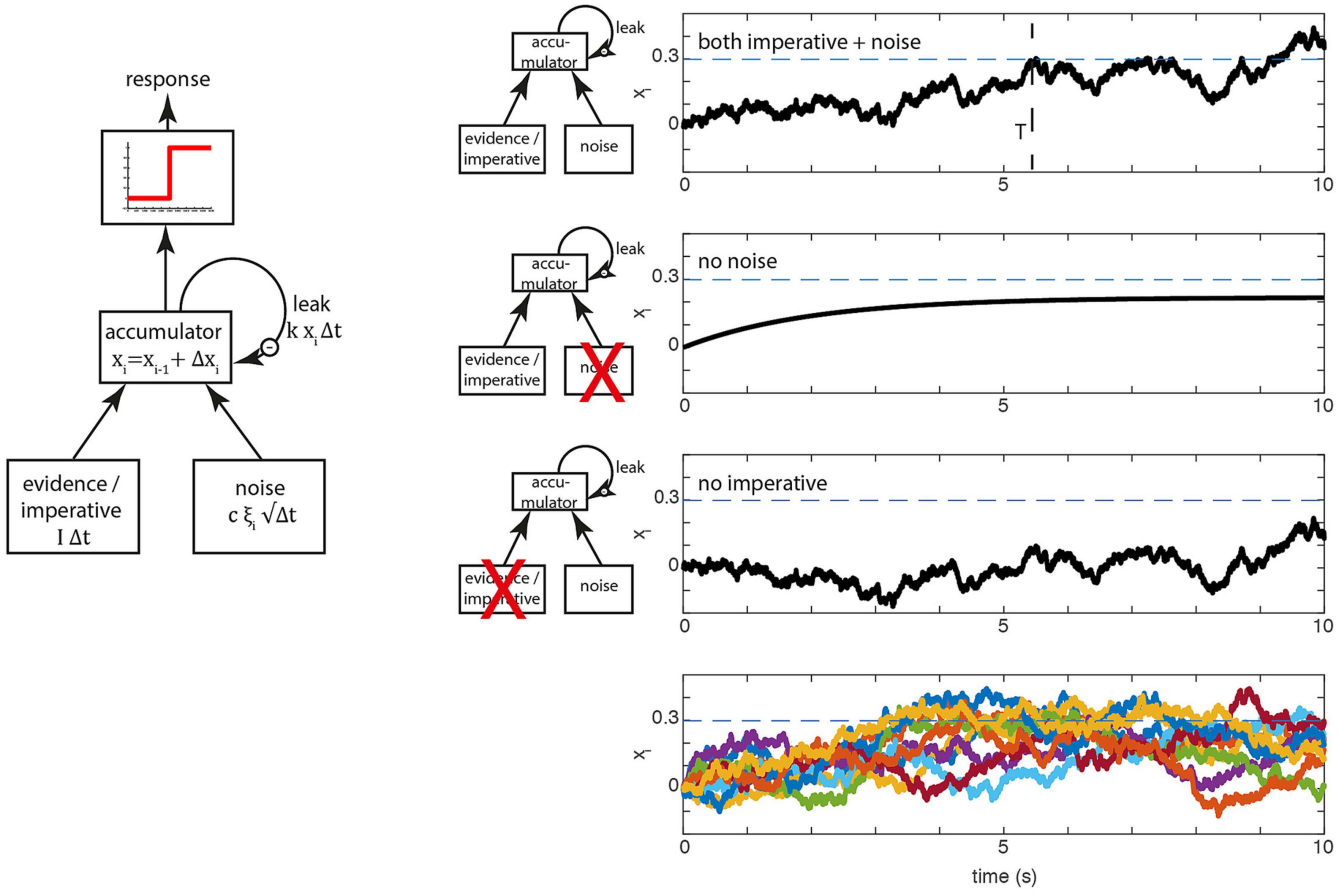
Basic accumulator model. (Left) In perceptual decision making at each time step, two variables, evidence and noise are added to a leaky accumulator. When the output of the accumulator reaches a certain threshold (red), a report is triggered. In spontaneous movement, the constant evidence is replaced by an constant imperative term that provides a strong constant input to the accumulator. (Right) Examples of the stochastic decision model (SDM; Schurger et al., 2012). The first three rows on the right show the behavior of the model in a single trial, separately for the full model (top), only the evidence/imperative and leak with noise removed (second row) and only the noise with leak (third row). The dashed horizontal line is the threshold 𝛽. The bottom row shows 10 trials, which clearly highlights the variability in individual trial accumulator trajectories. Note that in this original model, the imperative with leak will not drive the accumulator beyond the threshold, and the noise with leak will take an implausibly long time to drive the accumulator over the bound. Both terms together bring the signal across the threshold (at T, top row), which then triggers a movement with a distribution of reaction times that matches the waiting times of the participants until they press the button.

In this section, we will go into more detail about how the model works and which evidence is provided in support of it. We will focus on two papers, Schurger et al. (2012) and Schurger (2018), because these contain explicit mathematical formulations.^2^ The key variable is the accumulated signal *x*_i_ (Figure 1, left), often referred to as the “decision variable” in perceptual decision making. At the beginning of the trial this variable starts at *x*_0_ = 0 (a starting bias is not used) in their implementation. On every time step, an increment or decrement $\Delta x_{i}$ is added to $x_{i}$ and when $x_{i}$ reaches a threshold 𝛽 a movement is triggered at time point T also referred to as the waiting time (Please note: In a realistic brain, there is still a delay between the time T when the movement command is sent into the motor system, say down the spinal cord, and the time when the movement begins in the muscles, see Schurger et al., 2012).

At each time step, $x_{i}$ is updated by $\Delta x_{i}$ based on the following equation (rewritten from the original in a slightly more explicit form):


$$\Delta x_{i}=I\Delta t+c\xi_{i}\sqrt{\Delta t}-kx_{i}\Delta t$$

This means that the increment/decrement that is added to $x_{i}$ on each trial depends on three additive components:

1. The first term, IΔt, is a constant that is referred to as the “imperative” in the SDM. While this is mathematically equivalent to the (mean) evidence in accumulator models of perceptual decision making it has a rather different interpretation.
2. The second term, $c\xi_{i}\sqrt{\Delta t},$ reflects internal Gaussian noise $\xi_{i}$ that is scaled by c and $\sqrt{\Delta t}$ (in the SDM both Δt and *c* are fixed scaling constants).
3. The third term, $kx_{i}\Delta t,$ represents leakage, with the leak constant k scaled with another constant Δt. Thus, the accumulated signal is reduced by a constant proportion of $x_{i}$ on each time step.

When the accumulated signal $x_{i}$ crosses the threshold 𝛽, a motor command is triggered. Figure 1 (left) shows the operation of the model expressed as a more conventional box and arrows model. There, the three inputs from above are shown as arrows feeding into the accumulator. Let us look at the behavior of a single trial (Figure 1, top right; Figure 1, bottom right, shows this process for a large number 184 of trials). In every trial, the accumulation starts at the first step at $x_{0}=0$ (other accumulator models sometimes introduce a starting bias here). At every time step, the increment (or decrement) $\Delta x_{i}$ is added to $x_{i}$ , resulting in a noisy drift toward the decision boundary. At some point, the accumulated signal crosses the decision boundary (Figure 1, top right, dashed line) and triggers a response at latency T.

The next two rows of the figure show the consequences of removing the imperative (i.e., the constant) term vs. the noise term. If the noise term is removed, the signal $x_{i}$ rises and depending on the parameters of the model asymptotes below the threshold [as shown here, with the parameters in Schurger et al. (2012)] or it crosses the threshold (Schurger, 2018). If the imperative term is removed and the other variables are kept the same, the signal meanders around for a long time and at some point crosses the threshold, but with an implausibly long latency. Thus, in this variant of the model, both the imperative and the noise are involved in bringing the system to the threshold, primarily because the threshold is chosen based on the behavior of the full model (incorporating both the imperative and noise).

## Differences between the spontaneous motor decision model and perceptual decision making

Please note that the model employed by Schurger et al. is a simplified version with only a single accumulation process as opposed to multiple competing accumulators in Usher and McClelland (2001). Thus, the SDM reflects the absolute evidence for a single decision rather than relative evidence between multiple decision alternatives, as is frequently used in perceptual decision making. The SDM for endogenous decisions is a one-choice evidence accumulation model (Ratcliff and Van Dongen, 2011) with an added leakage term (Usher and McClelland, 2001). The two scenarios differ only regarding the interpretation of the parameter I . In perceptual decisions, the drift term I refers to the mean sensory evidence. Normally, in perceptual decision making, the constant term, the sensory evidence, is the main driving factor toward the decision boundary. The noise component is sometimes referred to as reflecting moment-to-moment *changes* in evidence. Note that the trial-by-trial variability of the response time is only affected by the noise term. When the constant term is zero, the behavior of a perceptual accumulator is governed by the noise term (see Figure 1, right).

We would like to highlight two points of the accumulator model in perceptual decision making. First, in the model as formulated here, for a given evidence level the drift I is a constant (see, e.g., Ratcliff, 1978, for variations on this assumption). It reflects the mean amount of sensory evidence that some neurons are encoding about an external stimulus property. For example, a random dot motion stimulus of a specific coherency level on an external monitor will involve a mean level of evidence with additional fluctuations in motion area MT in the brain, and this evidence is summed up by the accumulator. Second, the term “evidence” here means that the signal in MT has information *about another property*, the external motion stimulus. This evidence can also be very small or even 0 in case of very weak or no sensory evidence.

So how does this perceptual decision-making model transfer to spontaneous movements? In a review paper Schurger and colleagues state:

"*A strength of SDMs [stochastic decision models] is that they provide a unifying story that seamlessly allows agents to move between reason-driven and random decisions, as the spontaneous action case is *just an SDM driven by noise* in the absence of evidence/reasons.*" (Schurger et al., 2021, p. 10, underline added).

**Figure 2:**
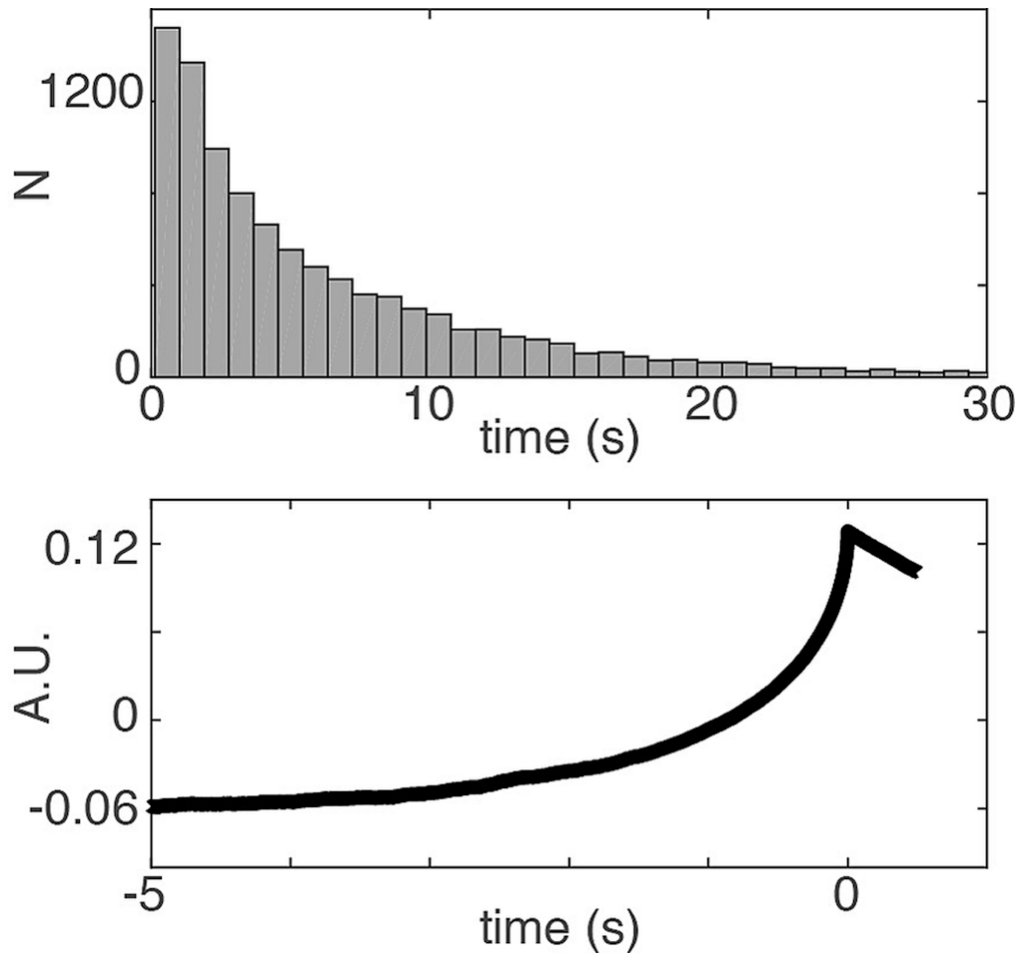
The distribution of waiting times (Top) in a variant of the Schurger et al. (2012) model where the imperative was set to 0 and the other parameters were adjusted (threshold was adapted to 0.1265 corresponding to the 80th percentile of the output amplitude, Schurger et al., 2012). With this adjustment, the distribution of waiting times does not match the shape of the empirical waiting times of subjects performing the task. However, the RP (Bottom) from that model has typical RP characteristics. Thus the imperative is important in order to fit the behavioral data (see also Guevara Erra et al., 2019). But even with zero imperative the SDM can explain the typical shape of the RP.

Based on this statement, one might think that the spontaneous movement model (SDM) is based on the perceptual decision-making model, but with zero constant evidence, with only the noise active, thus making it similar to perceptual guessing. It has indeed been shown previously that perceptual guesses (perceptual decisions with no sensory evidence) and spontaneous decisions indeed elicit similar activation patterns in posterior parietal cortex (Bode et al., 2013). Thus, it is also a reasonable hypothesis to explain free choices using an accumulator that is provided with only stochastic input but no evidence. However, in the accumulator model of Schurger et al. (2012), the constant drift/evidence term I was explicitly not set to zero, but was rededicated and given a new role as an “imperative” parameter. It was subsequently confirmed by the authors that the accumulation of sub-threshold noise alone would not be sufficient to fit the behavioral and brain data (see Figure 2; Guevara Erra et al., 2019).

The roles of imperative and noise in the model have been implied to reflect a two-stage sequential process:

“*In our model this solution amounts to simply shifting premotor activation up closer to the threshold for initiation of the instructed movement and waiting for a random threshold-crossing event.*” (Schurger et al., 2012, p. E2905).

**Figure 3:**
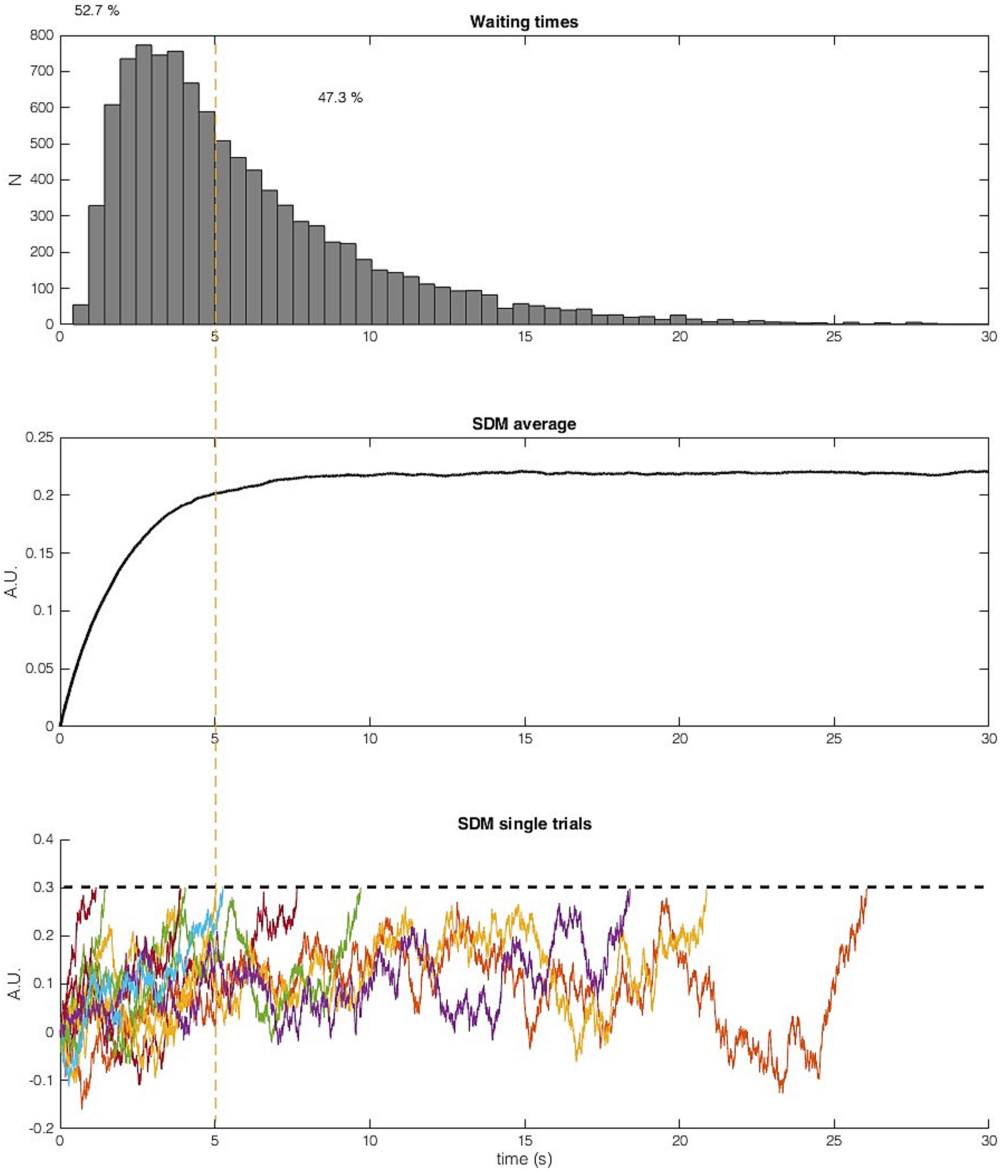
Result of 10,000 trials of the SDM calculated with the parameters reported in Schurger et al. (2012). (Top) Histogram of waiting times. (Middle) Average SDM output of 10,000 trials. The average SDM converges after around 5 s. In the averaged signal, the noise across trials cancels out and the result is similar to a SDM with only imperative and no noise as input (Figure 1). (Bottom) Ten sample trials of the SDM (truncated after crossing the threshold, dashed line). More than half of the trials (52.7%, left side of orange line) cross the threshold before 5 s. Trials with long waiting times do not stay close to the asymptote but fluctuate strongly. Neither trials with a waiting time faster than 5 s nor trials with a very slow waiting time shows the proposed behavior that the signal is moved closer to the threshold and that then some noise causes a threshold crossing. The imperative and noise both continuously influence the SDM signal, while the imperative is only driving the signal up, noise is driving the signal up and down.

We will see more examples of this notion below and demonstrate that the claims may be misinterpreted: the contribution of the imperative is substantial (see below). In the model, there are also no two discrete stages, in the sense that first there is a pre-stationary stage that moves the system toward threshold alone and then a stationary stage where the system waits for a threshold crossing. Half of the decisions happen during the early stage that is dominated by the imperative moving the signal closer to the threshold (see below and Figure 3).^3^ Thus, it might be better to speak of a two-component rather than a two-stage process.

## How is the accumulator linked to the readiness potential?

**Figure 4:**
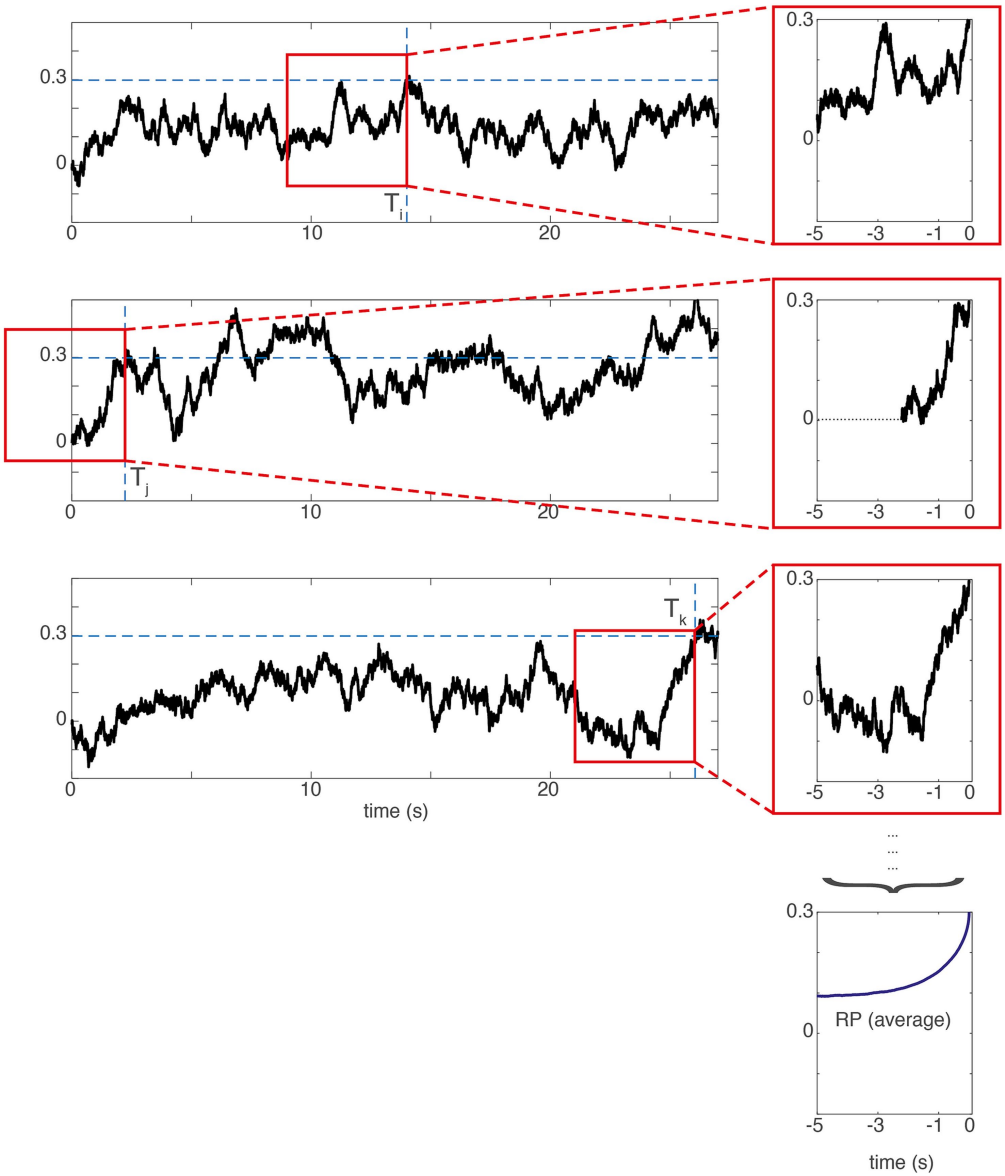
The SDM assumes that the readiness potential reflects individual trajectories of the accumulated signal *backwards*-averaged from the time of threshold crossing (T). The left shows artificially generated trajectories of a hypothetical accumulator signal $x_{i}$ in three different trials. The red box shows the 5 s averaging time window averaged backwards from the threshold crossing that can be seen on the rightmost border. The top three panels on the right show the signal in the red window enlarged and temporally aligned. The bottom right panel shows the average of these threshold-crossing-aligned trajectories across 1,000 trials. This curve can fit the shape of a readiness potential. When the threshold crossing happens early in the trial (second row), the missing values are left out in the average. The model fit is conducted at the level of the RP averaged across 1,000 trials. Also, see see Schurger (2018) for different assumptions underlying the spectral nature of these noise fluctuations and for different architectures of the model. Please note that the RP directly derived from the model is positive-going because the threshold is positive as in the original paper. There the time course is sign-reversed to match the empirical RP, which is a negative-going voltage deflection.

In order to provide support for the model, Schurger et al. (2012) show that it provides a potential explanation of the readiness potential. The idea is that the RP emerges from averaging the trajectory of the accumulated signal $x_{i}$ *backwards* from when it reaches the threshold (Figure 4). Importantly, all the fitting here is done based on the *average* RP, i.e., by averaging across many trials (for single-trial extraction of RPs see, e.g., Schultze-Kraft et al., 2016). Figure 4 shows this principle and plots some sample trajectories using the best fitting parameters from Schurger et al. (2012). These are obtained by fitting the waiting time distribution predicted by the model to the empirical waiting time distribution observed in the behavioral data. Please note that the waiting time distributions are subject to additional transformations before being compared (Schurger et al., 2012, p. E2906). Given those specific parameters the model also predicts the readiness potential. Also note that the readiness potential only reflects the final stage of the modeled decision-making process. Thus, a distinction has to be made about the claims made by the entire decision-making model and the claims made relating to predicting the shape of the readiness potential.

## Is there direct empirical evidence for the role of noise fluctuations in the RP?

Next we would like to address a number of confusions that seem to have originated in the literature on the SDM. For example, summaries provided in various papers give the impression that the empirical analysis of the EEG data and of behavioral waiting times provides *direct* evidence for the involvement of fluctuations in the decision process (see below). This is not the case. Instead, the fluctuation time series are latent and hypothetical variables of the model that are not directly measured (as is the case in many other neuroimaging models). The fluctuating time courses were not measured at the single-trial level.^4^ To date, there is no directly-measured evidence that stochastic fluctuation time courses generate the readiness potential. Note also that the scalp-measured-EEG will be affected by multiple, wide-spread fluctuations, most of which will not be directly related to the decision process. Thus, it is unlikely that it will be possible to establish a trial-by-trial link between the hypothetical fluctuations and the EEG signal.

Furthermore, in recent years, there have been substantial challenges to the ubiquity and nature of a core feature of the SDM, that is the accumulation process: Especially in situations where sensory evidence is brief rather than distributed across time, accumulation might not always take place (Thorpe et al., 1996; Uchida and Mainen, 2003), in other than in trivial ways (obviously one could debate whether, e.g., the superposition of postsynaptic potentials constitutes “accumulation”). Furthermore, the true dynamics of information processing during decision-making might be difficult to infer from average data (e.g., Latimer et al., 2015). One solution might be to obtain invasive recordings in human patients, insofar as possible (see below), as in Fried et al. (2011) (see below).

## Is the model supported by invasive recordings in animals?

In order to provide more direct evidence, the authors point to converging studies on animals. Potential evidence for the neural implementation of a SDM for endogenous tasks was reported by Murakami et al. (2014). That study investigates spontaneous movements in an intertemporal choice task in rats. After a go-signal, rats are given a choice between an immediate water reward or, if they wait for a delayed second signal, a much higher reward. Sometimes rats wait a bit, but then spontaneously abort and go for the smaller immediate reward. These choices on “impatient trials” are considered endogenous because there is no immediate trigger to move. For these trials they make two observations: (a) The activity in one selectively chosen population of neurons (P1) in rat motor area M2 rises sharply in the last few hundred ms before the movement. They interpret this as an accumulated evidence signal; (b) In a separate selected set of neurons (P2), some neurons (P3 ⊆ P2) show activity that is predictive of the waiting time early in the trial.

**Figure 5:**
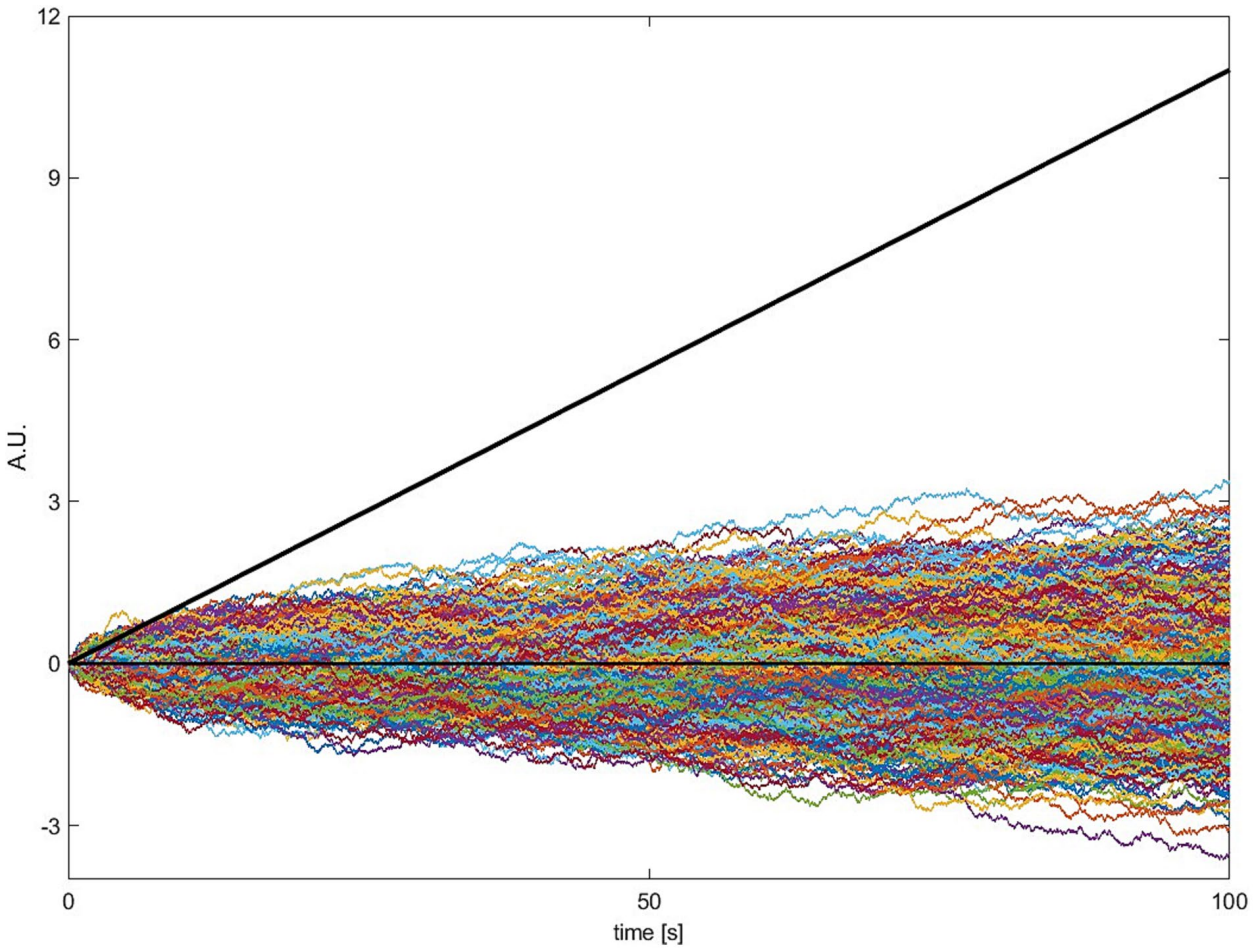
Simulation of a linear ballistic accumulator model (LBA). (Top) Waiting times generated with the LBA. (Middle) Schematic plot of the linear ballistic accumulator model. The starting position is drawn from a uniform distribution (between 0 and 4,000). The drift rate is drawn from a normal distribution (mean = 1, std. = 2). The threshold is at 6,000. (Bottom) Readiness potentials generated with the LBA (blue) and the original SDM (red). Please note that the RP of the LBA is scaled in order to match the RP of the PNAS model (Schurger et al., 2012). Please note that in order to match empirical RPs both models involve additional scaling factors, which also ensure that the polarity of the time course is inverted to match the polarity of the empirical RP.

**Figure 6:**
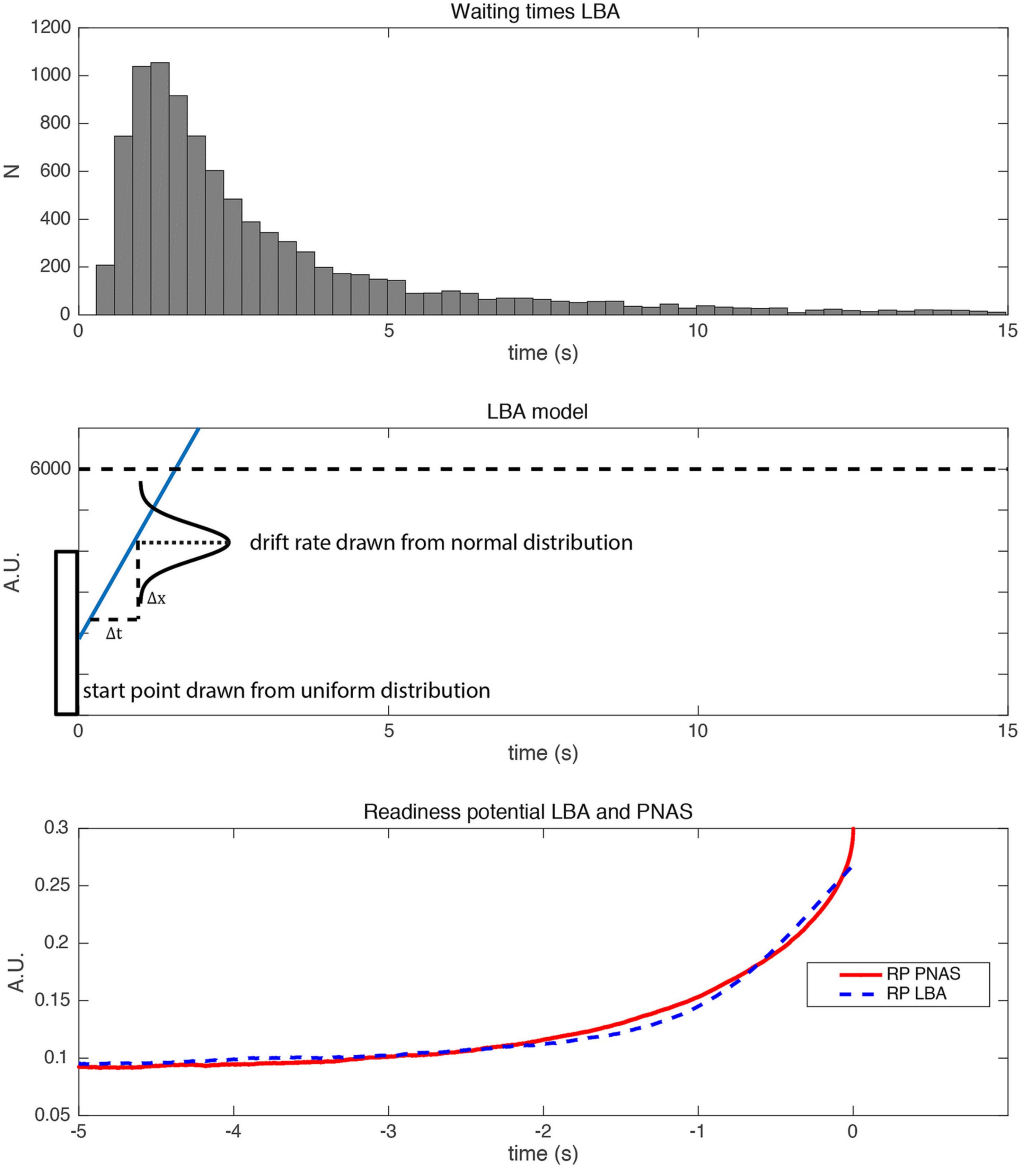
The cumulative input into the accumulator across the trial, plotted separately for stochastic and constant input. The cumulated imperative ( ∑IΔt ) is deterministic for every trial (bold black line) and thus there is only one trace. The cumulated noise ( $\sum c\xi_{i}\sqrt{\Delta t}$ , colored lines) is shown here for 1,000 different simulated trials. Please note that the cumulated noise across simulated trials is on average zero and the amplitude of single trials can be positive and negative. The noise is negatively correlated with the waiting time. Positive values will push the total signal over the threshold quicker whereas negative values will work against reaching the threshold and lead to longer waiting times. Most importantly, it can be easily seen that the cumulated imperative is almost always higher compared to the cumulated noise, especially for later time points.

At first sight, the similarities could be seen as providing support for the SDM. A careful look, however, shows that the superficial appearance of similarity may be misleading. By inspection of Figures 5C,D in Murakami et al. (2014), one can see that a *majority of predictive time periods are around the start of the trial or even before the onset of the trial*, which is the opposite of what would be expected in the case of an accumulator model. We will return to this later. Furthermore, the predictive signal is transient. Thus, this specific signal does not seem to map on to any variable of the SDM model, neither to a continuous stochastic or constant input, nor to an accumulator continuously integrating input across the trial. Instead, it could simply reflect a cognitively interpretable bias signal, such as an expectation on that trial of when the delayed reward will occur. If anything, a different subpopulation of neurons exhibits a ramping-like behavior, but mostly toward the end of the trial. Importantly, there is a considerable temporal dissociation between the time where most of the time windows are informative, and the time when the putative accumulator in their data ramps toward threshold (compare Murakami et al., 2014; Figures 5, 6). If in contrast, the predictive signal feeds into the accumulation one would expect it to appear close in time to the steepest increase in the accumulated signal. In their decision model, the signal from each contributing neuron is only collected in a single brief time window, and otherwise ignored (see their p. 1584). Thus, the accumulator model proposed by Murakami is quite different from conventional accumulation models.

## Are random fluctuations necessary in order to account for the data?

Given that the fluctuations have not been directly measured, but only indirectly inferred, it would be interesting to know whether a simpler model, potentially without fluctuations during the accumulation process, could in principle also explain the RP. Of particular interest would be a model that is compatible with the early, choice-predictive signals observed in the study by Murakami et al. (2014) mentioned in the previous section. We will here briefly present one such alternative model, the linear ballistic accumulator (LBA) that was originally developed for perceptual decision making (Brown and Heathcote, 2008). This does not have stochastic fluctuations during the trial, but replaces those with a randomly selected drift rate and starting bias that are only determined in a single step at the beginning of each trial. The LBA (Figure 5, middle) is a simplified version of the standard accumulator model.^5^ The difference is that the drift rate *I*, as well as a starting bias, is drawn from a random distribution only once at the beginning of each trial. Thus, it is not subject to noise fluctuations within the rest of a trial at all. It is thus an “early decision” model. The drift rate varies across trials, which could reflect, e.g., differences in attention (in the case of perceptual decision making) or differences in motivation or impulsivity (in the case of spontaneous movements). Interestingly, even out-of-the-box this fluctuation-free model makes very similar predictions for features of perceptual decisions to the accumulator. Importantly, it predicts the typical heavy-tailed reaction time/waiting time distribution. When used in a similar way to predict readiness potentials, the ballistic accumulator model also provides a good fit to the RP, despite its simplicity and the absence of random fluctuations during the trial (Figure 5). Please note that for this simple LBA model, the whole process is determined at the beginning of the trial. In the SDM, the exact time of a decision is left open and is subject to the fluctuations emerging throughout the trial (Schurger et al., 2012), thus constituting a “late-decision” model. Thus, in the absence of direct tests of the link between fluctuations and RPs, both early and late decision models appear to be equally plausible. For further discussion on the nature of “early” vs. “late” selection, please see the Appendix (“Early vs. late decisions”).

Please note that the LBA model also makes an important prediction that has been used as key evidence in justifying the SDM. When participants were interrupted by a click in the waiting period then the response to that click was faster when the EEG signal was more negative (Schurger et al., 2012). This is also predicted by the LBA. The more the signal has approached the threshold the shorter a motor reaction time would be if the accumulation processes for endogenous and exogenous movements share this common path.

## What are the relative contributions of “noise” and “imperative”?

In this section we will thus clarify the relative contributions of noise and imperative signals in the accumulation process. Summaries of the SDM frequently primarily focus on the fluctuations and largely ignore the constant component, beginning with the original paper:

“*One simple solution, given these instructions, is to apply the same accumulator-plus-threshold decision mechanism, but fed *solely* with internal physiological noise*” (Schurger et al., 2012, p. E2905; underline added).

In a subsequent paper the same authors say:

“[…] *when actions are initiated spontaneously rather than in response to a sensory cue, the process of integration to bound is dominated by ongoing stochastic fluctuations in neural activity* […]” (Schurger et al., 2016, p. 78, underline added).

“*In the case of spontaneous self-initiated movement there is no sensory evidence, so the process is *dominated by* internal noise*” (Schurger et al., 2016, p. 77, underline added).

And even later as we have seen above:

"*[*…*] the spontaneous action case is just an SDM driven by noise in the absence of* *evidence/reasons* " (Schurger et al., 2021, p. 10, underline added).

Subsequently, many other summaries of the findings ignore the role of the constant factor. For example, a subsequent version of the model, COINTOB, largely ignores this essential step as can be seen in their Figure 1 (Brass et al., 2019). They write:

“*[T]he threshold crossing is mainly determined by subthreshold neuronal noise [*…*]*” (Brass et al., 2019, p. 256, underline added).

“*A recent computational model [*…*] suggested instead that random fluctuations of a motor readiness signal could be sufficient to explain the initiation of voluntary actions[*…*]*” (Ganos et al., 2015, p. 52, underline added).

*“According to this model, the timing of the movement in the Libet experiment is determined by random threshold crossings in spontaneous fluctuations in neural activity. In particular, the model says that a decision when to move is determined by random threshold crossings only when it is not constrained by any evidence or reasons for action”* (Schlosser, 2019, underline added).

Note that all these assertions would suggest that the imperative is 0 or close to 0, which is not how it is actually modeled. As shown above, it is possible—at least in principle—to provide a reasonable fit of the RP with zero imperative (Figure 2). However, this does not provide a good fit for the decision times. It has been reported by the authors of the SDM that in order to obtain a good fit to *both* the waiting times *and* the shape of the RP within their model, the imperative is necessary (Guevara Erra et al., 2019). As we will see below, with the published SDM the imperative is not negligible, but an essential quantitative driving factor in threshold crossing.

If we look back to the perceptual decision-making case, the roles are quite clear. When the sensory information level is high then the accumulator primarily integrates this evidence throughout the trial (the component determined by *I* in the model above). When the sensory information level is absent, *I* is set to 0 and the behavior is driven purely by the noise. In the SDM with the published parameters, the situation is not like decision making without sensory information. *I* is not set to 0, so there is a constant driving input. However, as we have seen most of the interpretations in the literature focuses on the role of the noise fluctuations.

Some clarification is needed here, because the quantitative question of *how much* noise input versus constant input contribute to the crossing of the threshold can be dissected into three parts: First, as we have seen within the model [given the specific model parameters in Schurger et al. (2012)], *both are necessary conditions* for reaching the threshold in a realistic time window. Second, given that the imperative is a constant factor, it is clear that within the SDM framework the trial-by-trial variation in response time and the shape of the waiting time distribution are explained by the random component that is entailed in the noise input. This is very similar to accumulator models for PDM, where the trial-wise differences in decision times are explained alone by the noise component. It is thus trivially clear that within the SDM framework the trial-by-trial variation in response time can *only* be explained by the stochastic component that is entailed in the noise input.

Third, we can assess the *quantitative contribution* of each of the two inputs, constant imperative and variable noise, to the crossing of the threshold. The question here is: How much overall input have the constant versus the noise components provided at the point in time when the threshold is crossed. It is essential here to take a close look at the model. In each time step, the accumulator gets input from *both* the constant component (in the SDM the imperative) and the noise. Thus, movement toward the threshold is achieved by a combination of the continuous “push” of the constant and the variable (zero-mean) “rattle” of the random input. The combined and accumulated effect of these variables is additionally subject to a leak. One way to compare the contribution of the stochastic and the non-stochastic component to crossing of the threshold is to assess *how much of the distance the accumulator travels between zero and the threshold can be attributed to each component*. So we will sum up all the stepwise contributions of the noise component ( $c\xi_{i}\sqrt{\Delta t}$ ; i.e., the “rattle”) and also of the imperative component ( IΔt ; i.e., the “push”) separately up to the point where the accumulator crosses the threshold. This reflects the *input side* to the accumulator. Note that this is a stage prior to the leak (which in turn operates at the level of the combined accumulated signal, i.e., it affects the accumulated inputs of both noise and imperative).

A simple illustration of the net input of both sources is shown in Figure 6. It shows how much input to the accumulator has come from either the constant input (Figure 6, black line), i.e., the continuous “push,” vs the noise input (Figure 6, colored lines), i.e., the rattle. It is clear from Figure 6 that the accumulated input from the constant is much higher than that of the noise. Note that the colored curves in Figure 6 show the net accumulated input from (or integral of) the noise, not the noise itself. Note also that the time courses of the accumulated noise input wax and wane and are often below zero, as would be expected for zero mean noise.

**Figure 7:**
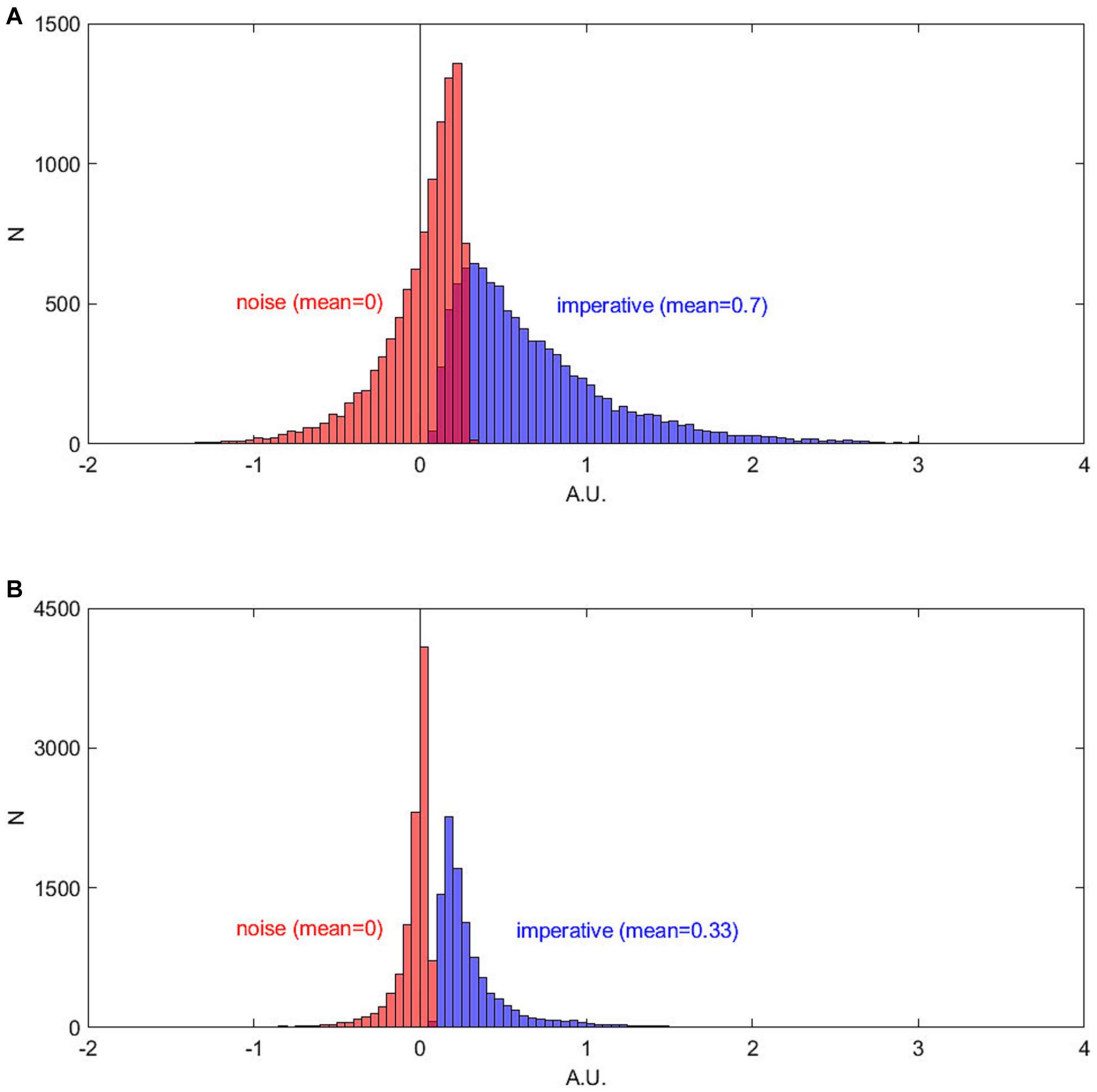
Total accumulated *absolute* contribution of imperative (blue, right histogram) and noise (red, left histogram) to the crossing of the threshold. The total contribution of imperative is always positive whereas the contribution of noise is in many trials even negative. The imperative dominates the overall input into the accumulator. **(A)** First version of the SDM (Schurger et al., 2012). **(B)** The finding is very similar for subsequent extended SDM (same scaling; Schurger, 2018). Please note that within this model the trial-wise differences in decision times are a separate matter. They are explained by the noise and not by the imperative (see text).

Figure 7 shows a different perspective on this process, now viewed *backwards* from the time of threshold crossing. The histograms show the cumulative input between trial onset and threshold crossing separately for the constant imperative and for the noise (and separately for the two published variants of the SDM). For the first SDM version (Schurger et al., 2012) the imperative dominates the net input. It has a mean accumulated input of 0.7 (arbitrary units, averaged across 10,000 trials). In contrast, the net contribution of the noise is on average 0 across all trials.

How is that possible? An analogy might help here: Let us assume a really strong person is pressing against a door to open it. They exert a strong force, but cannot quite get the door open. Then, a second, very weak person comes, does a very slight rattle on the door, whereupon the door jumps open. The combined force was sufficient to open the door, both are necessary. But the strong person makes the stronger quantitative contribution. It is similar for the SDM, where the constant push reflects the constant factor and the rattle is analogous to the noise. In the SDM, both factors are necessary, but quantitatively the constant factor makes the larger contribution [using the reported best fitting parameters from Schurger et al. (2012)].

Figure 7 reveals that in many trials the net contribution of the noise is even negative. But how is it possible that the noise can have a negative net input if it is at the same time necessary to bring the accumulated signal cross the threshold? This happens when the noise is negative for long stretches of the trial and thus cancels out the positive input from the constant imperative. Then, toward the end of the trial, a small positive contribution can help the accumulated signal over the threshold (see also below for more details). Thus, in these trials the noise *prevented* the threshold from being crossed early (Figure 7A). For a second version of the SDM (Schurger, 2018, with pink noise as input), the findings are very similar. The imperative dominates the input with a mean of 0.33 (averaged across 10,000 trials) and the noise contributes on average 0 (Figure 7B). The smaller values for the second model (Schurger, 2018) are due to a lower threshold compared to the first model (Schurger et al., 2012; 0.298 vs. 0.1256).

The relative strength of the noise and imperative obviously play a role here. We assessed this further by varying the relative strengths systematically (Appendix, “Relative strengths” and Supplementary Figure A2). Noise only dominates the accumulated input for very small values of I close to 0 (i.e., where the red curve is above the blue curve). Trivially, when I = 0, the noise provides the only input and this hence has to be positive. It is also clear that the average contribution of the noise across a wide range of parameters is close to 0 (Supplementary Figure A2), which is what would be expected for Gaussian noise with a mean of 0. Interestingly, the net noise contribution is negatively correlated with the waiting time. On short trials, the noise contribution is positive. That is because the constant input alone will not have provided enough net input and thus short trials can only occur when the noise also has a positive input. In contrast, long trials can only come about if the constant positive input of the imperative is counteracted by a net negative contribution of the noise (i.e., otherwise the threshold would have been crossed earlier). Thus, the longer the trial the lower the accumulated net noise contribution for crossing the threshold.

The relative contribution of imperative vs. noise to threshold crossing is also different for slow and fast trials. Long trials occur when the noise counteracts the positive input from the imperative, and then only just before threshold crossing the noise provides a positive input. This raises the question how much the noise vs. the constant input contribute to crossing the threshold in the last seconds before threshold crossing. This is also important because the SDM assumes that the readiness potential only emerges from the signals in the last 5 s of the time series. To address this, we will consider the quantitative contribution of noise versus constant in this final buildup period of the RP. We know that the noise has to have a positive contribution in that small time window. But does the noise input dominate over the constant input *at least in this final brief time window*?

**Figure 8:**
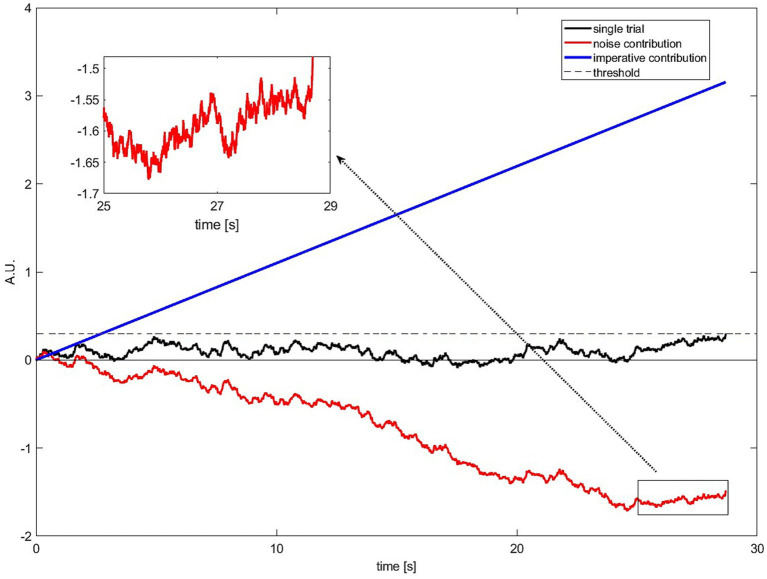
A single illustrative simulated trial of the SDM (black). The threshold is crossed relatively late at 28.7 s. The imperative contribution is constantly increasing and always “pushing” toward the threshold (blue). The noise contribution is negative going and counteracting the constant input, leading to a long waiting time for this simulated trial (red). The noise fluctuates (“rattles”) and there are small epochs during which the noise is pushing toward the threshold although on average it is negative going. Only at the very end of the simulated trial, there is a short time window in which the noise has a contribution that is strong enough to push the SDM across the threshold together with the imperative.

To illustrate this, we show one selected simulated and exemplary trial of the SDM for illustrative purposes (Figure 8). In the Appendix (“Quantitative contributions for final buildup”), we show mathematically that the following is also true for the averaged model RP. In Figure 8, we plot one simulated trial of the SDM with a long waiting time. For this trial, we also show how much the noise (red) and imperative (blue) contributes from 0 at the trial start to the threshold crossing. A long waiting time can only be the result of a weak or even negative contribution of noise because the imperative is a constant positive input. As can be seen in Figure 8, the total input of noise (red line) is strongly negative going. Within this negative going noise, there are small epochs in which the noise is slightly positive going. However, in most cases, this is not enough positive contribution so that the SDM crosses the threshold. Only at the very end of the trial there is a small time window in which the noise can, together with the imperative, contribute enough, such that the threshold is crossed. Please note that in half of the trials the noise contribution is positive going (see Figure 6). However, the general behavior is the same that the noise has the strongest positive contribution only very briefly before the threshold is crossed (for more details see the analyses in the Appendix).

However, one might consider our emphasis on the importance of the imperative a distraction. Why is this so important? There are important reasons to highlight the role of the imperative: in the model the imperative characterizes a fully deterministic component of the decision that is fixed once the trial has begun. The data by Murakami et al. (2014) also suggest that the information about when the decision is made is already there early in the trial. Furthermore, the LBA model where all the decisions are made at the beginning of the trial also predicts the RP and the distribution of waiting times (Brown and Heathcote, 2008). Thus, these facts can dramatically change the interpretation about whether the decision is made early or late, which was a key motivation of the SDM. Furthermore, there is a common view that the deterministic component is simply a preparatory stage, that brings the signal within reach of the threshold and then subsequently fluctuations take over. We will see in the following that this is also not the case.

## Does the “imperative” (constant) term first bring the system into a dynamic range where random fluctuations take control?

As we have seen above there is another important aspect of the model, that noise- and imperative-related processes are interpreted as constituting separable and *sequential* stages (for examples see above). The idea there would be that the constant signal initially drives a “*stochastic exponential transition period*” (Schurger et al., 2012, p. E2906) that first brings the accumulated signal into an operating range, and subsequently the fluctuations determine when the signal crosses the threshold (Schurger et al., 2012, p. E2906). Here are a few examples of this point:

“*After a stochastic exponential transition period [*…*], the leaky accumulator generates noisy trajectories whose threshold crossings determine movement times*” (Schurger et al., 2012, p. E2906);

“*In our model this solution amounts to simply shifting premotor activation up closer to the threshold for initiation of the instructed movement and waiting for a random threshold-crossing even*t” (Schurger et al., 2012, p. E2905);

“*According to their stochastic decision model, the decision process, given Libet’s instructions, amounts to simply shifting premotor activation up closer to the threshold for initiation of the movement and waiting for a random threshold-crossing fluctuation in RP*” (Bayne and Pacherie, 2015, p. 224).

Considering these statements, we should expect two effects: First, the accumulator is moved closer to the threshold without any (or only few) decisions being made. Second, from this plateau the system waits for a random threshold-crossing event.

Let us consider the time point 5 s into the trial where the accumulator has on average reached around 90% of its asymptote (see Figure 3, middle). One might assume that hardly any decisions have been made by this point, but quite the opposite is the case. In 52.7% of the trials, the threshold is crossed and a decision is made earlier than 5 s. For the trials with long waiting times, it can even be observed that the signal fluctuates strongly and sometimes even reaches negative values after it was first closer to the threshold (see the orange and yellow curves on the right of Figure 3, bottom). Therefore, the “move signal closer to threshold” process can even happen multiple times in slow trials. Thus, the verbal description and interpretation in the quotation above of noise and imperative does not capture the model behavior appropriately. During the entire time course of a trial both imperative and noise contribute to the current state of the accumulator. It is a concert of the two, both contributing to the process, with the imperative exerting a larger overall quantitative contribution toward reaching the threshold, and the noise explaining trial-wise differences in decision time. Please note, that above arguments also hold if time points earlier than 5 s are considered as reaching a lenient interpretation of a plateau. We observe many early decisions and the SDM signals fluctuate strongly.

Please note, in the extended version of the SDM with pink instead of white noise as input (Schurger, 2018), the logic of the two stages is even more problematic. There, the constant imperative can drive the SDM across the threshold alone, i.e., without the noise, and thus two sequential stages are not necessary anymore. Here is the proof: we can calculate $x_{i}$ at the asymptote, i.e., when $\Delta x_{i}$ is zero. We also consider the case without noise.


$$\Delta x_{i}=I\Delta t+c\xi_{i}\sqrt{\Delta t}-kx_{i}\Delta t$$

$$0=I\Delta t+0-kx_{i}\Delta t$$

$$kx_{i}\Delta t=I\Delta t$$

$$x_{i}=\frac{I}{k}$$

With the reported parameters (*I* = 0.1, *k* = 0.6 and threshold = 0.1256) the SDM would converge to 0.1/0.6 = 0.167 based on imperative alone and without noise. The threshold in this model is at 0.1256 so the threshold would be crossed without any noise in the model. Taken together, as mentioned above, it is not accurate to think of the SDM as a two-stage model, with phase 1 being “climb closer to threshold (but do not cross it yet),” and phase 2 being “OK, now you may cross the threshold at any time.”

## Is any “evidence” involved in the model?

In perceptual decision making, “evidence” refers to one variable having information *about* another, such as a perceptual representation having evidence about an external stimulus. This is the reason it is called “evidence” and not simply “a signal in MT” or “bias.” In contrast, in the SDM the imperative describes an intrinsic signal that is not evidence, but a signal that is necessary for the total accumulated signal to cross the threshold in behaviorally realistic times (Guevara Erra et al., 2019). In line with this, the authors of an animal study on endogenous movement decisions that they consider to reflect an accumulation process (Murakami et al., 2014), say that their task involves “*no evidence per se*” (p. 1580).

There seems to be some confusion in the literature about whether one of the signals (imperative *or* noise) might reflect evidence in the SDM after all. Already an early review paper interpreted the original study as

“*showing that bounded-integration processes, which involve the accumulation of noisy evidence until a decision threshold is reached, offer a coherent and plausible explanation for the apparent pre-movement build-up of neuronal activity*” (Schurger et al., 2016, p. 77, underline added).

Brass et al. (2019) interpret the original paper on the SDM as a solution that:

“*treat[s] stochastic noise in the motor system as evidence for the accumulation process*” (Brass et al., 2019, p. 256, underline added).

They then continue:

“*In contrast to perceptual decision making, however, the accumulation of evidence [in the SDM] is not based on perceptual information but on internal information and stochastic neural activity*”(p. 259, underline added).

And then:

“*These models assume that decision time in the Libet task is based on a process of accumulation of evidence to a threshold, just like in other decision-tasks. Because the decision is not based on perceptual or other external evidence, this accumulation of evidence might operate primarily on stochastic neural fluctuations in the motor system*” (p. 257, underline added).

And finally:

“*This means that the RP and the LRP do not reflect a ballistic process that necessarily leads to action but rather a gathering of evidence*” (p. 259, underline added).

Thus, it appears that some authors consider that the noise plays a role of evidence, and that there is some additional signal involved, here termed “internal information.” Also in other papers, there is some ambiguity as to the respective roles of the variable factor (i.e., the noise fluctuations) and the constant factor (evidence/imperative):

“*Schurger et al. propose that the motor system constantly undergoes random fluctuations of RPs and that this random premotor activity is used as a substitute for actual evidence*” (Pacherie, 2014, p. 36).

Of course, it is possible to go beyond the original formulation of the SDM and re-consider the imperative signal as having some computational function dedicated to representing decision-relevant internal states (such as motivation or impulsivity). One possibility could be that there is a single overall mechanism, but with two different types of input, one being sensory evidence and the other being imperative.

When considering one variable having evidence about another one would want it to fulfill some additional requirements. For example, the evidence should be able to “stand in” as a proxy of what it is representing (Shea, 2018, p, 15–16, 143). To illustrate this, we may turn to Brass et al. (2019), who note that it could indeed be sensible to assume that some latent internal signals could influence the buildup of the imperative when no external information is available. In such cases, the level of imperative could be somewhat constrained by these causally influencing factors, but we would not necessarily see the imperative as having a function of reliably tracking such variables and serving as a stand-in (i.e., “evidence”) for those. Not every causal influence can be considered as evidence. Please further note that if the imperative indeed played a role of collecting evidence it would also have needed to receive much more attention as an integral part of the movement decision, and not be largely ignored as we have seen above.

## Summary and outlook

The aim of this paper was to assess the level of evidence for the SDM and to address certain confusions that have arisen in the literature. First, we highlighted that there is no direct evidence based on neuronal-level measurements for the role of stochastic fluctuations in the RP and movement initiation and that the analyses are based on macroscopic signals averaged across many trials. This does not rule out the SDM as a model, but it clarifies what kind of data will be required to definitively validate the model. Second, we found that the purported evidence for the SDM from animal studies is limited and may not favor the SDM over other models. Third, we showed that a quasi-deterministic model where the parameters are fixed at the beginning of the trial (the LBA) makes very similar predictions to the SDM, including for the interrupted version of the Libet task, and fits well with a population of neurons found in an invasive monkey study. Fourth, we found that the literature has tended to ignore the deterministic component of the SDM, the imperative signal that accounts for an important part of signal input toward the threshold. Both the stochastic fluctuations and the imperative are necessary for reaching the threshold in realistic time periods with the published parameters of the SDM. Fifth, there is a confusion regarding the link between perceptual decision making and spontaneous movements. We have argued that the SDM is not just a special case of perceptual decision making, but without the evidence. Although mathematically identical to a leaky stochastic accumulator used to model perceptual decision making, the SDM does not incorporate any “evidence” *per se* (as assumed in some secondary sources, see above). In the context of the SDM, the constant (or mean) component of the “evidence” in perceptual decision-making is replaced by a different constant factor, an imperative to move given by the demand characteristics of the task. To avoid confusion, we recommend using the term “imperative” in the context of the SDM rather than “evidence.” Sixth, we remind the reader that a key aim of the SDM, to provide a pre-decisional account of the RP, cannot be fully addressed by the model because this hinges critically on the nature of the noise fluctuations as being “objectively random” versus “epistemically random.” This of course is true of any scientific model that incorporates randomness, and may be very difficult to decide empirically, but at least the case is far from closed.

There are some other questions that need to be addressed: Where is the ramp? The strong contribution of the constant (imperative) predicts a large initial rise of the accumulator signal at the beginning of the trial. This would predict a very stereotypical ramp at the beginning of the trial in the corresponding brain signals. In fact, invasive recordings from human single neurons have revealed a slow gradual ramping up of the signal prior to the time of decision (Fried et al., 2011).

Could the ramp not be the buildup of the intention? The fact that the imperative signal plays such an important role in bringing about the decision could point to a re-interpretation of the components of the SDM model. One way would be to interpret the imperative signal as the largely deterministic and gradual buildup of the intention to move (Schurger et al., 2016) and the smaller effect of randomness some form of intrinsic variability. Would this mean that the decision is made early or late? Random variability from trial to trial is observed in just about any task (from threshold perception to motor performance) without this randomness necessarily being considered the most relevant property of the process.

It could also be useful to extend the scope from thinking about spontaneous movements in general to what happens specifically in spontaneous movement *experiments*. These lab experiments impose constraints that are not present in real-world free-ranging actions. For example, there is an explicit or implicit affordance to move within a reasonable time-frame (e.g., to not wait too long) and at the same time avoid being predictable or rhythmic, which Schurger et al. (2012) refer to as the demand characteristics of the task and incorporate in the model as the imperative (or drift) term in the SDM. Already the earliest paper on readiness potentials stated: “*The participant was required to perform the movement not rhythmically, but in irregular intervals*” (Kornhuber and Deecke, 1965, p. 1, our translation). In the study by Schurger et al. (2012), the instructions are to “*[*…*] try not to decide or plan in advance when to press the button, but to make the event as spontaneous and capricious as possible.*” (Schurger et al., 2012, p. E2911). What if the participant is thinking: “Oh dear, am I spontaneous or capricious enough?,” and if so what would they do? In the classic Libet study, the participants are required to “[…] *let the urge to act appear on its own at any time without any preplanning or concentration on when to act*” (Libet et al., 1983, p. 625). One might wonder what participants were thinking if they did not experience such a mysterious “urge.” Would they have just waited for the whole duration of the experiment and then finished the experiment by saying: “*Sorry, but I never felt an urge to move*”? As has been pointed out previously (Schurger et al., 2012, Supporting Information) the key point here is that the preparation of these movements might have involved a vast array of cognitive processes, conscious or unconscious. These could include (among others) mental time keeping and time-based prospective memory (McDaniels and Einstein, 2000), inhibition of behavioral impulses to move immediately (Noorani and Carpenter, 2017), or generation of random behavior sequences (Nickerson, 2002). Sticking with the latter, obviously, in order to be random and capricious one could in principle use a random time interval generator based on the accumulation of fluctuations. But why generate or use a long series of random numbers, if all you have to do is generate one single random number at the beginning of the trial (as, e.g., in the LBA)? Please note that the LBA model is arguably no less parsimonious than the SDM because it involves fewer variables (i.e., it does without the unmeasured and thus hypothetical fluctuation time series). We would like to clarify that we do not want to argue that the verdict is already in for an early decision model (as in the LBA), or that the SDM can be ruled out based on the evidence. At the current state of evidence, the debate between early-decision and late-decision accounts is still not settled. We point out that the empirical support for the model is currently not yet definitive, and that also several key conceptual issues still need clarification. We hope we have provided some of that clarification here.

## Data availability statement

The raw data supporting the conclusions of this article will be made available by the authors, without undue reservation.

## Ethics statement

Ethical approval was not required for the study involving humans in accordance with the local legislation and institutional requirements. Written informed consent to participate in this study was not required from the participants or the participants' legal guardians/next of kin in accordance with the national legislation and the institutional requirements.

## Author contributions

CB: Conceptualization, Formal analysis, Investigation, Methodology, Visualization, Writing – original draft, Writing – review & editing. BG: Conceptualization, Investigation, Writing – review & editing. J-DH: Conceptualization, Funding acquisition, Project administration, Resources, Supervision, Writing – original draft, Writing – review & editing.

## Footnotes

^1^A leaky accumulator is similar in some ways to a low-pass filter and generates similar slow fluctuations when provided with noisy input (Eliasmith and Anderson, 2004).

^2^They are both similar variants of the larger family of accumulator models (e.g., Smith and Ratcliff, 2004). We will reorder some aspects for easier readability, and we will use difference equations rather than their differential variants because all their modeling is done in discrete time steps *i*.

^3^Please note that an imperative signal is only one way to bring the accumulated signal and the threshold closer together. Alternatives could potentially be changing the starting point of the accumulation process or the decision boundary itself (but see Guevara Erra et al., 2019) or progressively “collapsing” the decision boundary across time (Hawkins et al., 2015).

^4^The gap between model variables (e.g., noise time courses of hypothetical units) and measured variables (EEG signals) is not unusual in modeling of neuroimaging data. For example dynamic causal modeling (DCM; Friston et al., 2003) also models measured data using a number of latent variables at finer levels of temporal resolution. Note that there the model is used to explain raw time series data, not averages.

^5^In fact, one could question whether the LBA really is an “accumulator” at all, despite providing a good fit to the data and carrying the label “accumulator” in its name. The answer to “when the decision is actually made” is very different for the LBA than for the SDM, because the key factor that determines the outcome is present already at the beginning of the trial. As mentioned above, this model is somewhat more compatible with the data by Murakami et al. (2014) because it predicts that the response time is encoded in brain signals already at the beginning of the trial.
