# Supplementary material for: Clarifying the nature of stochastic fluctuations and accumulation processes in spontaneous movements
Source: Front Psychol. 2023 Oct 12;14:1271180. doi: 10.3389/fpsyg.2023.1271180 (PMC10602783; doi:10.3389/fpsyg.2023.1271180)
Supplement: Supplementary file 1 [file Presentation_1.pdf]

Appendix

Early vs. late decisions

As mentioned above, a classical interpretation of the readiness potential is that it reflects a *post*-decisional stage of processing after an unconscious decision to act has been made (Schurger et al., 2012). First the brain makes an *early* decision, then a process (of which the RP is an indicator) is triggered that prepares the movement. Some consider this to be counterintuitive because of the long temporal delay of many hundreds of milliseconds between the brain's unconscious decision and the time when a participant consciously believes to be "making the decision now" (Libet et al., 1983). An important reason for the interest in the stochastic decision model is that it seems to remove this counterintuitive time delay. It implies instead that the decision to act occurs *late*, that is when the accumulated signal crosses the threshold for action (Brass et al., 2019; Schurger et al., 2012), which is much closer to the subjective time of decision. Everything happening before that is pre-decisional activity in the brain (some of it stochastic, according to the SDM). In this view the readiness potential originates at a *pre*-decisional stage and is an artefact of averaging stochastic signals aligned to the time of a threshold crossing.

Just as a reminder, "decision" here can mean two things: (a) the participant's conscious experience of making a decision, and (b) some (potentially unconscious) brain event, quasi a "neural decision"[1], that somehow sets the brain on the track for executing the movement (these two events may or may not coincide in time). Because the discussion of the SDM has focused on the threshold crossing, which is a property of the neural system, we will focus on neural decisions and set the problem of subjective decisions aside. And in order to avoid a too extended general discussion of the role of randomness and (in)determinism in biological systems, we will base our discussion on properties of the computational models. The question we want to discuss here is whether accumulator models always imply late decisions. We would like to suggest that the interpretation of the *nature of the fluctuations* in the SDM is vital when interpreting the decision as early or late. Please note that this is not specific to the SDM though, but it holds for any stochastic accumulator model.

In the papers on the SDM the fluctuations are described as "internal physiological noise" or "random fluctuations" (Schurger et al., 2012, p. E2905 and E2904). This could mean different things, so an important distinction is required. **(a)** Either this could mean that they are "*objectively random*" in the sense of the randomness being an irreducible part of the world that would not be predictable in more

[1] We use the term "decision" here without further discussion, but consider a clarification of what could constitute a decision without making reference to the subjective experience a major challenge in this field. From a neural level of resolution, brain processes can be described as trajectories in a high-dimensional state-space. It is unclear what would constitute a decision along such a trajectory. If one considers phase transitions or bifurcations as decisions, as criteria then these would be ubiquitous.

detail however much we learn about the world. An extreme version of this were if fluctuations were guided by quantum processes (analogous to e.g. radioactive decay)[2]. Due to their indeterminism such fluctuations could not be predicted. Whatever one might find out about the states of the world, there is an intrinsic indeterminism that remains. **(b)** On the other hand the randomness could refer to "*epistemic randomness*", where a signal would *appear* to have random properties, but this would reflect the fact that some properties of the process are not known (e.g. due to insufficient data or to not understanding the algorithm, say as in the case of a deterministic random number generator). The process could also be unpredictable despite being deterministic (as for example in deterministic chaos). The randomness might also constitute a mixture of **(a)** and **(b)**, so we might uncover more latent determinants as research progresses, but there still remains an irreducible remainder.

Now let us see whether the interpretation of the nature of the processes leading up to the threshold influences the time at which we would consider the decision to happen. In order to cleanse our thinking of the a-priori assumptions we have with accumulator models, let us consider a physical analogy, a Rube Goldberg-style simple chain reaction model of a ball rolling down a slope towards a row of dominos (Fig. A1). The dominos fall over one by one and then ring a bell. In each trial the ball is set off with a slightly different speed and we measure the time until the bell is rung. Note that when the ball knocks over the first domino stone a nonlinearity is reached, such that a different process is triggered. If the ball for some reason were prevented from reaching that stage, say it were to mysteriously move uphill again, then the chain reaction in the dominos would not be triggered. Thus, knocking over the first domino is similar to passing a nonlinear threshold (analogous to β).

We will consider four different cases: **(1)** In the first case (Fig. A1, top) a ball rolls down a constant *smooth* slope, knocks over the first domino, which triggers a predictable chain of events until the last domino rings the bell. Everything in this model is perfectly determined at the beginning of the trial when the ball is set off with a certain speed. **(2)** In the second case (Fig. A1, middle) everything is the same, with the exception that the surface is now rough. Let's assume that the rough surface causes the ball to bump back and forth on the way, thus adding a random component to the time it takes to pass the ramp. Let's further assume that the effect of these bumps is indeterministic in principle, so that however precise we can measure the ball and the surface there would still remain some degree of indeterminacy. **(3)** Now let's consider a third case, similar to (2) but now the effect of the bumps is perfectly deterministic. When the ball is let go with a certain speed and the same rough surface the run time is always the same. **(4)** Let's also consider a variant on (3). Now in every trial we use a different rough slope (Fig. A1, middle and bottom). Each of these slopes is perfectly deterministic, but we don't

[2] At least in indeterministic interpretations of quantum mechanics.

know which one is chosen on a given trial. Case 1 approximately symbolizes the LBA, case 2, 3 and 4 represent variants of the SDM.

Cases 1 and 3 are clearly deterministic. Case 2 is indeterministic. And case 4 is concealed determinism that appears as indeterminism because a latent variable (which slope is chosen) is not known. The rolling down the slope is an analogy to the drift phase and the domino stones are an analogy for the motor execution stage triggered after passing the threshold. If all the relevant causal factors are established (as in cases 1, 3 and 4), isn't the outcome of the decision then pre-determined and it can thus be considered already made? It would be interesting to conceptually clarify this issue for interpreting the implication of stochastic processes in general.

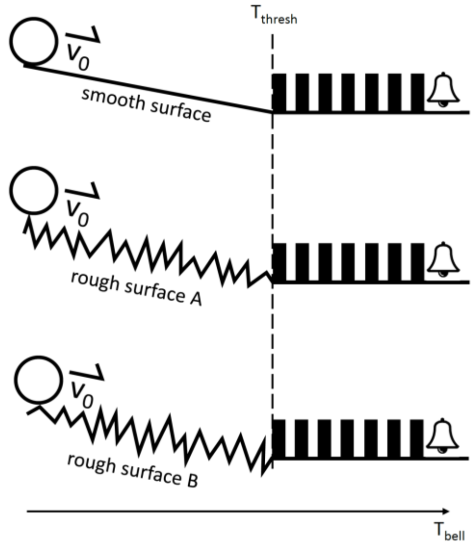

Figure A1: Simple causal chains inspired by Rube Goldberg to illustrate the models. **Top:** A ball is set off on an even slope with variable speed v_0 that is determined at the beginning of the trial from a Gaussian random distribution. The slope is chosen to exactly balance the friction and keep the ball at a constant speed without acceleration or deceleration. At the end of the slope at time T_{thresh} the ball triggers a chain of dominos that runs deterministically through and then sounds a bell at time T_{bell}. Note that if the ball (for some reason) were to be stopped from reaching the dominos they would not be triggered, so the passing of the threshold can be considered a nonlinear event. When would the decision be made when the ball reaches the bell? There is only one free variable: the speed with which the ball is initially set off. So is the decision made at the beginning of the trial? **Middle:** Now the same causal chain is set off but with an added rough surface. Now the time that is needed to pass the rough slope influences the time until the bell is rung. But is this process deterministic? That depends on the nature of the rough surface. One type of surface would potentially result in slightly different bumps on every trial and thus add some irreducible variability to the run time. A different type of surface might still potentially yield reproducible run times. **Bottom:** Now, let's stick with the reproducible version and exchange the rough slope on a trial-by-trial fashion, each time with a different reproducible slope. Let's assume we don't know which slope has been picked on a given trial. When is the decision about the run time made? Given that following the release of the ball (analogous to the beginning of a trial) everything is fully deterministic, one plausible interpretation would be to say that the decision is made

at the beginning of the trial, despite appearing to depend on random processes on the way. Even though the model only reproduces some properties of the accumulator models (for example there is no leak) it can help form our intuitions about which events might count as decision points. Without an in-depth understanding of the nature of the noise and without an ability to measure it, it is impossible to say whether a process is deterministic or not. And whether the decision is early or late will presumably depend on the answer to this question.

Relative strengths

To assess how the relative strengths of imperative and noise affect their relative contributions to the accumulator we repeated the simulation for different values of the constant factor I while keeping the noise scaling factor c and all other parameters constant (from Schurger et al. 2012) (see Fig. A2). At the time of reaching the threshold, the total input contributing to the accumulator is much higher from the constant imperative than from the accumulated noise for a wide range of values of I (Fig. A2, accumulated imperative = blue curve; accumulated noise = red curve).

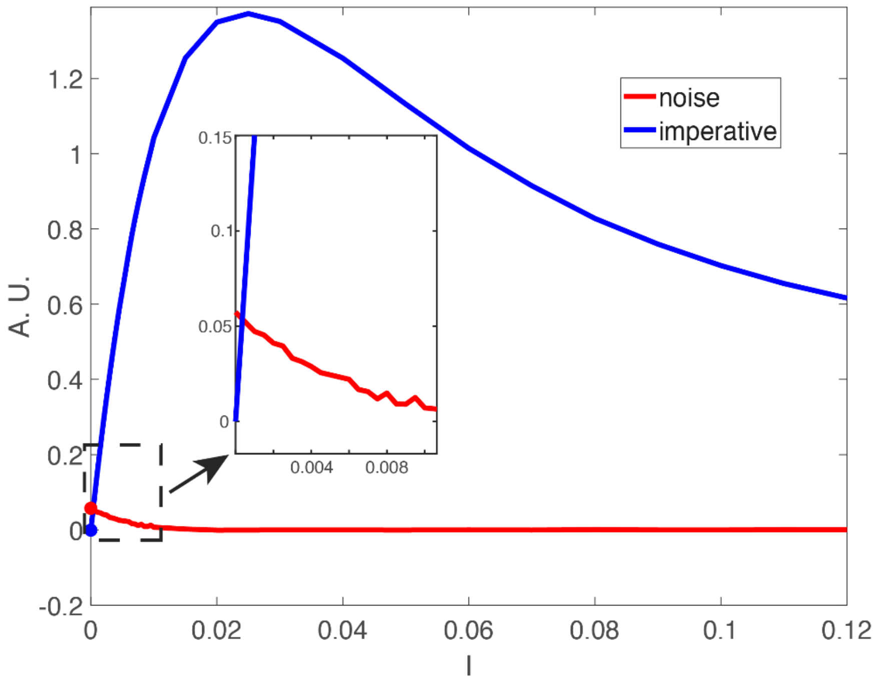

Figure A2: Total averaged accumulated *absolute* contribution of imperative (blue) and noise (red) to crossing the threshold for different values of *I* (imperative) in the model (all other parameters are constant and are taken from Schurger et al. 2012). For most values of *I* the accumulated total contribution of *I* exceeds the accumulated total contribution of the noise. Only for very small values of *I* close to 0 the contribution of noise exceeds the contribution of imperative. This is when the imperative is so small it hardly contributes to the drift towards the threshold (which could be achieved alternatively with noise with much higher variance). Note that the accumulated contribution of *I* rises first and then decreases again (with *I*). This is because the waiting times for low values of *I* are much

longer and thus the amount of accumulated signal lost to the leak is higher. For higher values of *I* the waiting times are shorter, so the amount of accumulated signal lost to leak is smaller and thus the overall input is smaller. Please note that the contribution of the noise to the explanation of the trial-wise differences in decision times is a separate matter (see text). Also note that due to the leak (that affects the combined input of both sources) the total accumulated input has to be much higher than the threshold value.

Quantitative contributions for final buildup

Here we will calculate the average quantitative contribution of noise versus constant in this final buildup period of the RP. We know that the noise will typically have a positive contribution in that small time window. But does the noise input dominate over the constant input *at least in this final brief time window*?

To address this, we will consider the model RP resulting from back averaging from the threshold crossing event in 1000 trials (see above main text). We separate the model RP into its 3 additive components (imperative, noise and leak) based on the core equation of the SDM, repeated here for convenience:

$$\Delta x_i \; = \; I\Delta t \; + \; c\xi_i\sqrt{\Delta t} \; - \; kx_i\Delta t$$

Please note that we can use the equation also for the average model RP and not just single trials. From the given model RP we can calculate average Δx_i. We also know x_i (which is the model RP at time step i) and thus can calculate the average leak ($kx_i\Delta t$). Then we can calculate the average noise contribution because the imperative is constant. By repeating this calculation for every time step of the model RP we can separate the different contributions. We separated the model RP starting from 3 different time points (-2, -1 and -0.5 s). Finally, we cumulated the 3 different components from the starting points (-2, -1 and -0.5 s) to 0 s (when the threshold is crossed) (see Fig. A3, top row). Please note that we extend this analysis and look at the contribution of the average leak as well because the model RP is the sum of all the three components.

Please note that this is a biased analysis regarding the average noise contribution. Just before crossing the threshold the noise must have a positive contribution. Thus, although the noise contribution across the whole trial might be negative in a single simulated trial (see above) in a small time window before crossing the threshold the noise contribution has to be positive. Consequently, it is not surprising due to the selection of the time windows before averaging, that the average noise has a positive contribution. However, it can be seen that for the longest of the three time windows (-2 to 0 s) the imperative still dominates the input (Fig. A3 top left). Only for the shortest time window (-0.5 to 0 s) the average noise clearly dominates the input over the imperative (0.11 vs. 0.06 respectively) (Fig. A3, top right).

In a next analysis we calculated two alternative model RPs for which we set either the imperative or the noise to 0 at three different time points (-2, -1 and -0.5 s). We used the average noise estimates from the first analysis and recalculated the leak according to the equation of the model. With this analysis we simulated how the model RP would change if one of the 2 main input sources would have been switched off in order to illustrate their role for crossing the threshold and for the shape of the model RP (Fig. A3, bottom row). The leak in the model is relatively strong (see Fig. A3, top row purple). Therefore, switching off the constant input of the imperative leads to a strong decrease of the alternative model RP at first (Fig. A3, bottow left, yellow). This further shows how small the input of the average noise is, as it cannot compensate for the pull of the average leak down to zero, until roughly 500 ms before the threshold is crossed when the alternative model RP without imperative still increases. It is also very important to note that even for the shortest time window (Fig. A3, right) in which the average noise contributes more than the imperative, the average noise is not strong enough to push the accumulator over the threshold (0.298) without the constant push of the imperative. In other words, switching the imperative off 500 ms before the decision would not have generated a decision (with the reported parameters). Please note that the noise plotted in Figure A3 is the average noise and therefore it appears smooth without the typical "rattle".

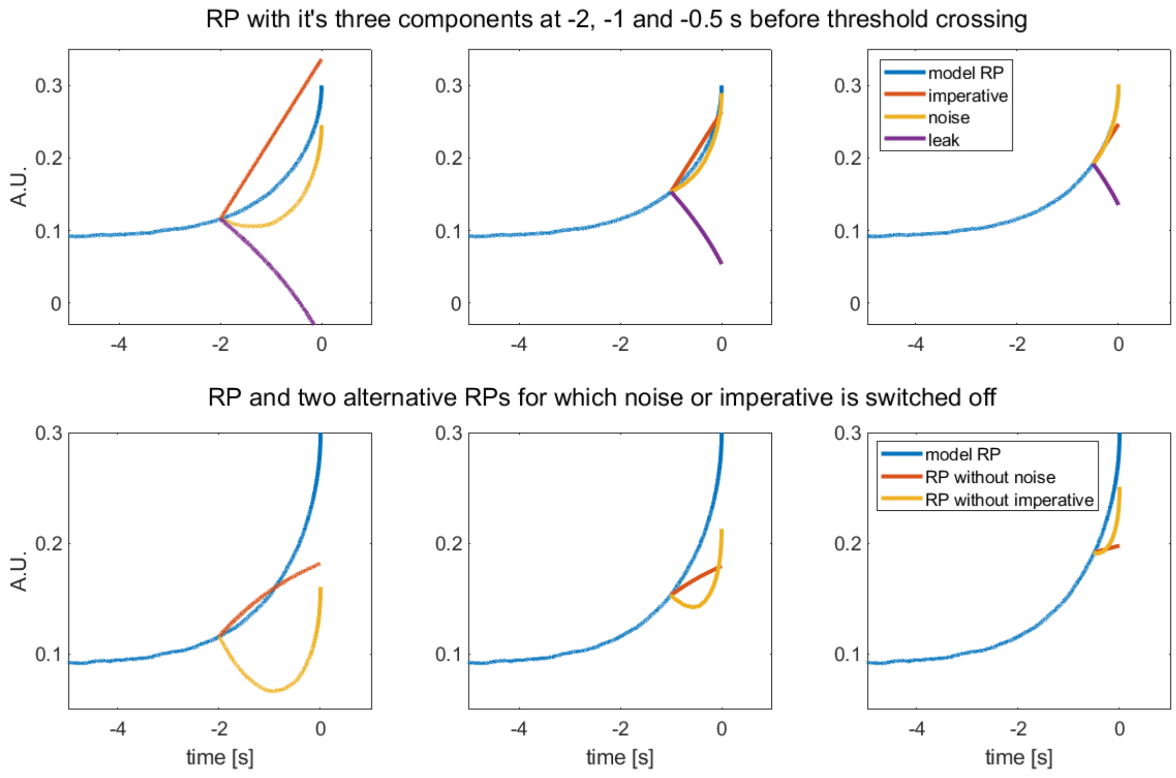

Figure A3: Top: Model RP (blue) and the separated contribution of imperative (red), noise (yellow) and leak (purple) into the model RP for 3 time windows (-2 to 0, -1 to 0 and -0.5 to 0 s from left to right). The contribution of imperative:leak for the 3 time windows is 0.22:0.13, 0.11:0.14 and 0.06:0.11. The later and shorter the time window the higher the

relative contribution of noise (compared to imperative) with a higher contribution of noise compared to imperative for the shorter time windows. Bottom: RP (blue) and hypothetical alternative versions of the RP for which the noise (red) or imperative (yellow) input was set to 0 at -2, -1 or -0.5 s (from left to right) before crossing the threshold. None of these hypothetical alternative RPs would have crossed the threshold of 0.298. Even for the shortest time window of 500 ms in which the average noise contributes roughly twice as much compared to the imperative, the constant input is needed to cross the threshold.

References

Brass, M., Furstenberg, A., & Mele, A. R. (2019). Why neuroscience does not disprove free will. *Neuroscience & Biobehavioral Reviews*, *102*, 251–263. https://doi.org/10.1016/j.neubiorev.2019.04.024

Libet, B., Gleason, C. A., Wright, E. W., & Pearl, D. K. (1983). Time of conscious intention to act in relation to onset of cerebral activity (readiness-potential). The unconscious initiation of a freely voluntary act. *Brain: A Journal of Neurology*, *106 (Pt 3)*, 623–642. https://doi.org/10.1093/brain/106.3.623

Schurger, A., Sitt, J. D., & Dehaene, S. (2012). An accumulator model for spontaneous neural activity prior to self-initiated movement. *Proceedings of the National Academy of Sciences*, *109*(42), E2904–E2913. https://doi.org/10.1073/pnas.1210467109
